# Impact of air-polishing using erythritol on surface roughness and substance loss in dental hard tissue: An *ex vivo* study

**DOI:** 10.1371/journal.pone.0286672

**Published:** 2024-02-26

**Authors:** Anne B. Kruse, Stephan Fortmeier, Kirstin Vach, Elmar Hellwig, Petra Ratka-Krüger, Nadine Schlueter

**Affiliations:** 1 Faculty of Medicine and Medical Center, Department of Operative Dentistry and Periodontology, University of Freiburg, Freiburg, Germany; 2 Faculty of Medicine and Medical Center, Institute of Medical Biometry and Statistics, University of Freiburg, Freiburg, Germany; 3 Hannover Medical School, Department of Conservative Dentistry, Periodontology and Preventive Dentistry, Hannover, Germany; Universidade de Trás-os-Montes e Alto Douro: Universidade de Tras-os-Montes e Alto Douro, PORTUGAL

## Abstract

This *ex vivo* study aimed to investigate surface roughness and substance loss after treatment with different professional cleaning methods and to determine whether subsequent polishing with a rubber cup and polishing paste is necessary. Samples (flat and natural surfaces) of human enamel and dentin were prepared (baseline) and treated with either a curette, air-polishing with erythritol, a rubber cup and polishing paste, or a combination thereof (treatment). Subsequently, all samples were immersed in an ultrasonic bath (ultrasonic) to remove residues from the treatment procedures. The surface roughness values sRa and sRz as well as tissue loss were measured profilometrically. Linear regression models were used to compare group differences (roughness and loss) considering the corresponding baseline value. The significance level was set at *p<0*.*05*. sRa increased significantly after treatment with curettes or air-polishing with erythritol in both enamel (*p<0*.*001*) and dentin (*p<0*.*001*) of flat samples. The same effect was observed for sRz in dentin (*p<0*.*001*) but not for enamel compared to negative control. Polishing with a rubber cup and paste alone had no significant effect on roughness values. When combined with other treatments, the effect of curette or air-polishing with erythritol dominated the effect. In enamel, none of the tested methods led to measurable tissue loss. In dentin, air-polishing with erythritol caused ≤50% tissue loss compared to the curette. Conclusively, for enamel, treatment effects on roughness were measurable but of limited clinical relevance. For dentin, air-polishing resulted in a smaller but insignificant roughness increase and less tissue loss compared to the curette. Polishing with a rubber cup and paste did not affect surface roughness. Regarding the clinical application, the use of air-polishing seems to be a less invasive procedure than using a curette; polishing with rubber cup and paste offers no advantage in terms of reducing roughness as a final procedure.

## Introduction

Air-polishing is considered an efficient and gentle cleaning procedure for the removal of extrinsic stains and accumulated biofilm from supragingival dental hard tissues [1]. For this reason, it is often performed as an integral part of professional dental cleaning and periodontal therapy. However, the literature on the effects of air-polishing on dental hard tissues is mainly limited to *in vitro* studies. Some authors found that air-polishing does not lead to clinically relevant roughening of the enamel, and subsequent polishing with a rubber cup and polishing paste is therefore unnecessary [2,3]. Other studies, however, report significant abrasion of dental hard tissues depending on the powder type used [4]. Low-abrasive powders such as glycine or erythritol left significantly less roughness after application to enamel and dentin than sodium bicarbonate and other higher-abrasive powders [2]. Therefore, for dental hard tissues with a low surface hardness, such as demineralized enamel, dentin, or exposed root surfaces, only low-abrasive powders are recommended for air-polishing [5]. While the latter aspect has been answered in *in vitro* studies, the impact of a single cleaning aid normally used for professional tooth cleaning and the combination of the various tools on surface roughness and tissue loss has not been investigated thus far. In addition, further research is needed to answer the question of whether a classic polish with a rubber cup and polishing paste after the use of air-polishing with low-abrasive powders leaves a less rough surface. Of particular interest in this context is whether a polish leads to reduced surface roughness through abrasion or whether the polishing paste merely masks the roughness by remaining in grooves and pits.

Therefore, the aim of this *ex vivo* study was to investigate the effect of different supragingival cleaning methods routinely used for professional tooth cleaning (curette, air-polishing, rubber cup and polishing paste) and their combinations on the parameters of surface roughness and substance loss of dental hard tissue (enamel and dentin). The null hypothesis was that there are no differences in surface roughness and tissue loss after treatment with different cleaning methods.

## Material and methods

**Fig 1:**
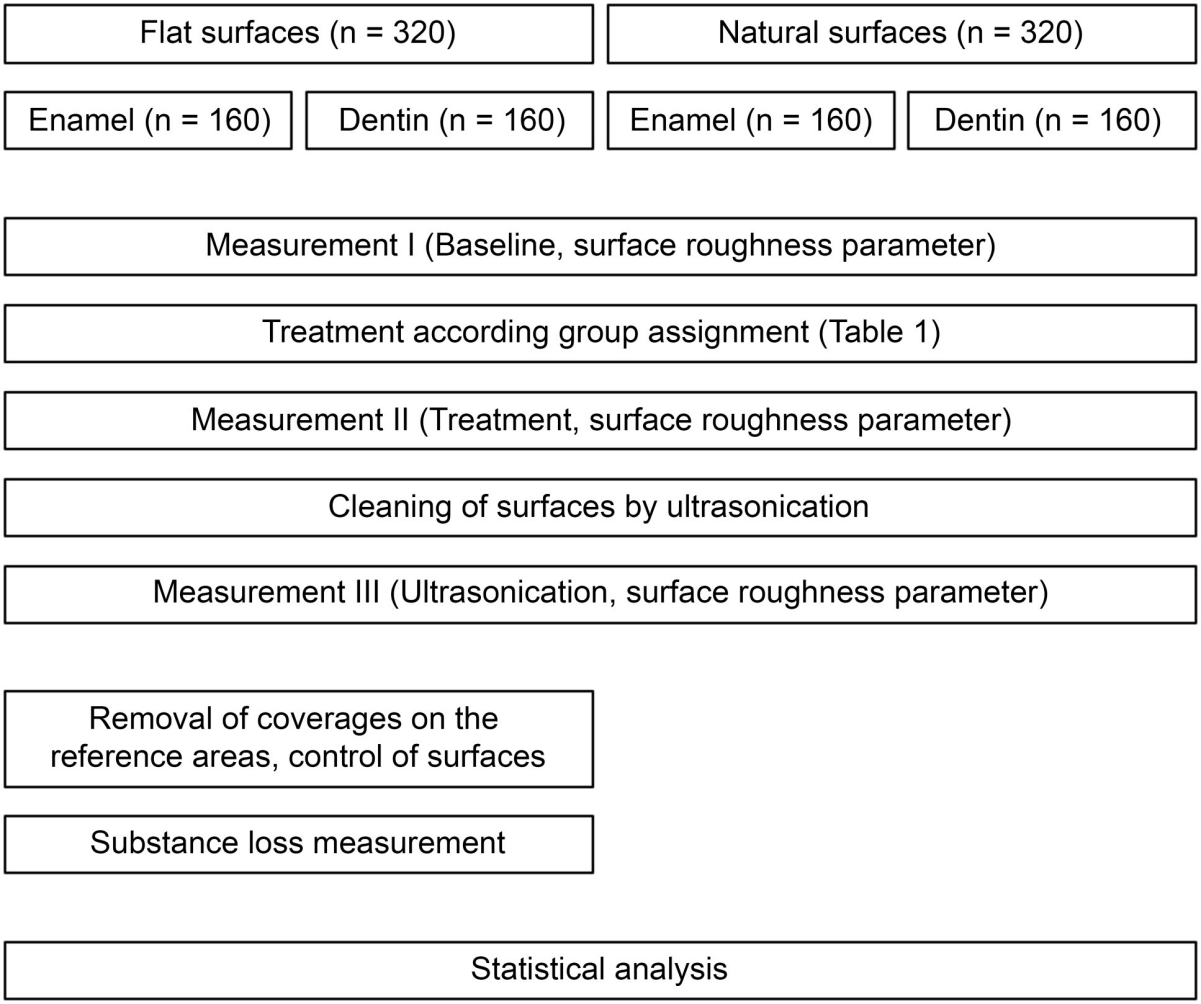
Flow chart of study procedures.

This *in vitro* study consisted of four different substrate-surface combinations with two different substrates (enamel and dentin) and two different surfaces per substrate (flat surfaces and natural surfaces). A total of 640 samples (160 for each substrate-surface combination) were prepared from extracted human teeth in a standardized manner. Sample preparation was followed by incubation in pooled human saliva to obtain a pellicle. Afterward, various combinations of different cleaning methods (scaler, rubber cup, air-polishing; Table 1) were applied to the samples, followed by an analysis of the surface changes (for details see flow chart in Fig 1). Sample preparation, treatment, and measurement procedures were standardized, and all performed by the same trained technical examiner (SF).

**Table 1:** Group assignment according to treatment procedures.

Group	Treatment		
	Curette	Air-polishing	Rubber cup
1 –Curette	1.		
2 –Air-polishing		1.	
3 –Rubber cup			1.
4 –Curette/air-polishing	1.	2.	
5 –Curette/rubber cup	1.		2.
6 –Air-polishing/rubber cup		1.	2.
7 –Combination of three	1.	2.	3.
8 –Negative control (only pellicle)			

Numbers indicate the order of treatment.

### Sample preparation

The dimension of the single samples, both for flat and natural samples, was defined during preliminary experiments in order to allow for a sufficient cleaning and measurement procedure.

#### Flat samples—Enamel, Dentin

Sample preparation was carried out according to a previously applied scheme [6]. The human enamel and dentin samples were obtained from human teeth extracted in dental practices in Germany and anonymously used with the patient’s verbal informed consent for scientific purposes at the Albert-Ludwigs-University in Freiburg (approval of the study by the Ethics Committee of the Albert Ludwig University Freiburg, No.469/19). After the extraction, teeth were stored in a saturated aqueous thymol solution (Thymol, Fluka Chemie AG, Buchs, Switzerland).

For flat enamel samples, the root was removed from the teeth, and the enamel of the smooth surfaces was cut longitudinally (Exakt cutting system, Exact Apparatebau, Norderstedt, Germany). For flat dentin samples, the remaining dentin around the pulp was also longitudinally cut without opening the pulp chamber. The side of the dentin facing away from the pulp was marked and used to ensure maximum homogeneity of the samples. The resulting enamel and dentin slices were ground flat and polished in a standardized way (Exakt micro grinding system, Exakt Apparatebau, Norderstedt, Germany). For enamel, the natural outer surface was removed; for dentin, the side facing away from the pulp was polished. All surfaces were ground with P1200 sanding sheets (Exakt Apparatebau, Norderstedt, Germany). The polishing of the samples was carried out with abrasive foils (P2500, Exakt Apparatebau, Norderstedt, Germany; 3 μm, Microdiamant, Lengwil, Switzerland). All cutting and grinding procedures were performed under constant water-cooling (min. 50 ml/min). The standardized polishing procedure resulted in a defined surface quality, removal of approximately 200–250 μm of the outer enamel, and produced a sample surface of at least 3 mm x 3 mm. All samples were checked for impurities, damage, and cracks (Leica Wild M3Z microscope, 6.5x to 16x magnification) and sorted out if necessary. In total, 160 enamel samples and 160 dentin samples were prepared according to the sample size calculation (see below). These were mounted in pairs on glass slides using Technovit 7230 VLC (Kulzer-Exact, Wehrheim, Germany) to ensure uniform treatment with saliva. Subsequently, markings were applied to the glass surface, which enabled a reproducible measurement of the surfaces. Then, half of the sample surface was covered with scotch tape to create a reference area to be able to measure potential substance loss. The uncovered area of the sample surface served as the test area. The samples were stored at 4°C at 100% humidity (humid chamber) until further use.

#### Samples with natural surface—Enamel, Dentin

Teeth were cleaned by removing soft tissue with a scalpel. Teeth with macroscopic defects due to for example extraction procedures and areas with calculus or concrements on the surfaces were discarded. The crown was separated from the roots. Afterward, the crowns were divided into two halves. For enamel samples, the cut side was mounted to a glass slide. For dentin, the roots detached from the teeth were divided in such a way that the outer curvature was preserved and mounted with the cut side to a glass slide. All samples were mounted in pairs of two on one slide. Like mentioned for the flat surfaces, marks were then made to the glass surface to enable reproducible measurement.

#### Saliva collection and incubation of samples

All samples were incubated in pooled human saliva. Saliva collection was performed according to a previous study that was already approved (Ethics Committee of the Albert Ludwig University Freiburg, No. 135/18) and approved by the vote for the present study (Ethics Committee of the Albert Ludwig University Freiburg, No.469/19). For the present study, only stimulated saliva was used. Stimulation was performed mechanically by chewing on paraffin wax pieces. Saliva was collected in Falcon tubes. A saliva volume of 3.5 ml was calculated per sample, which corresponds to 70 ml per group and substrate; therefore, a total of 3.2 L was necessary. Saliva was collected from 23 individuals, of which a maximum of one-third was either female or male donors. The saliva was then centrifuged and disinfected with a 10% sodium azide solution, resulting in a concentration of 0.2% sodium azide. The slides with samples were incubated for two hours in saliva at 36°C in a water bath with shaking movements (35/min linear) to ensure sufficient pellicle formation.

#### Treatment procedures

After saliva incubation, the samples of Groups 1–7 within each substrate-surface combination were treated with the respective cleaning methods or their combination in the order indicated in Table 1. Procedures were based on clinical treatment procedures so cleaning with air-polishing using erythritol powder, mechanical cleaning with a curette, polishing with a rubber cup and polishing paste, as well as the combination of two or three of the methods, were investigated.

**Fig 2:**
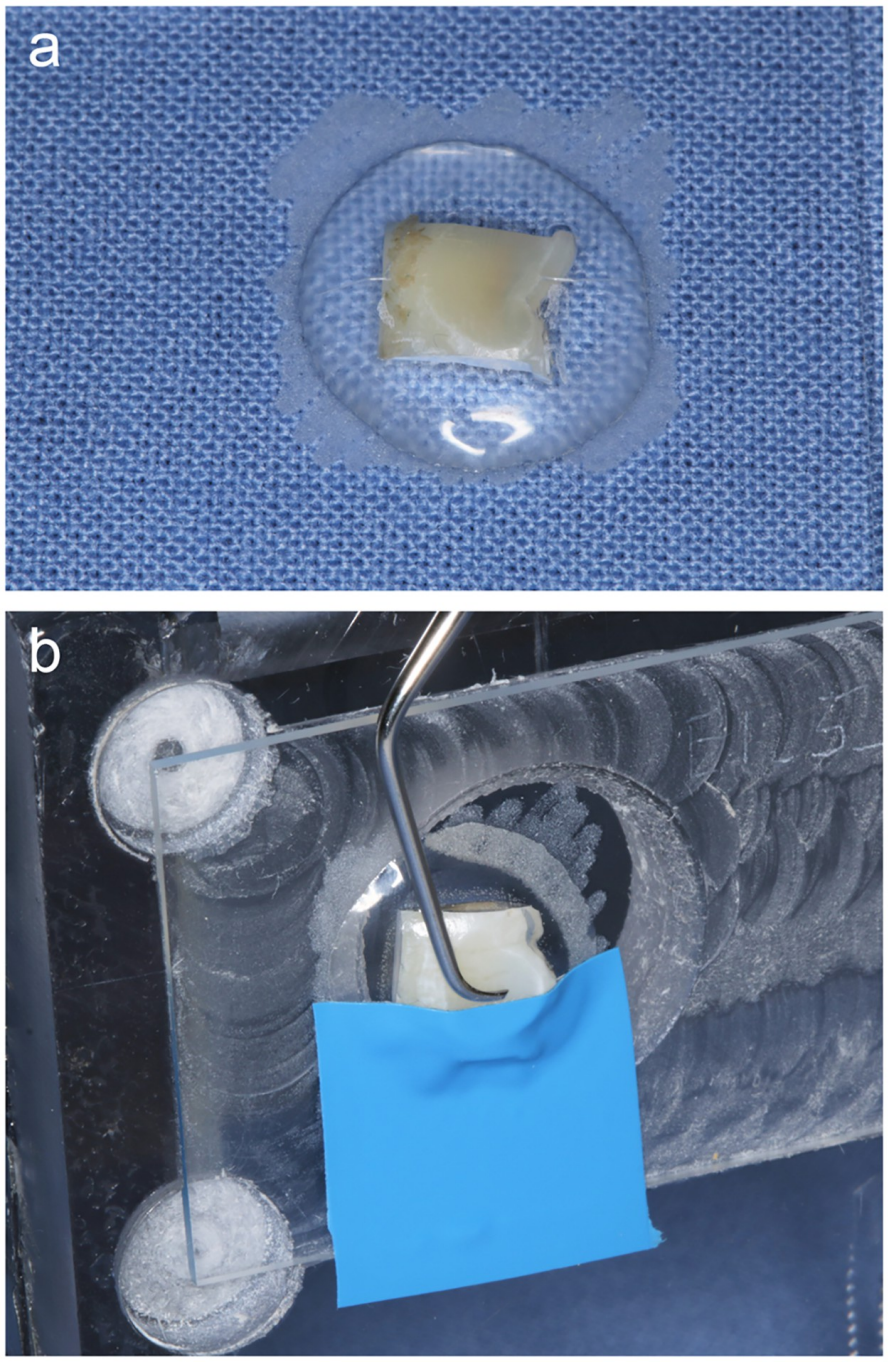
Example for a flat enamel sample. (a) Sample fixed on a glas slide with light curing acrylic resin. (b) Glas slide with flat enamel sample mounted to the bench. The surface was half covered with a tape to create a reference area that allows loss measurement. The tape was removed prior to loss measurement. The fixation of the sample on the bench allows using a standardized angle with the curette or the air-polisher.

*Treatment with curette*. The sample surfaces were instrumented by a trained examiner (SF) using a curette (Gracey #7/8 oral/labial, green; Hu-Friedy, Frankfurt am Main, Germany). Instrumentation was carried out performing 10 strokes in a specific, previously defined direction, which was marked on the sample slide. A special bench was used to ensure that the angle of instrumentation was always the same to increase the homogeneity of the treatment (Fig 2). The examiner was trained to use always the same pressure (0.491 N). The calibration was performed by using a letter scale. During perfomance of the study, a continuous recalibration was performed in order to ensure constant treatment conditions. For further standardization, the curette used was resharpened after treatment of five samples, each.

*Air-polishing*. Air-polishing was performed for 5 seconds by a trained examiner (SF) using an air-polishing standalone unit (AIRFLOW^®^ ONE, EMS Dental, Nyon, Switzerland) and erythritol powder (EMS PLUS powder, EMS Dental, Nyon, Switzerland) with the setting water 10, powder 3 under constant movement (dynamic pressure 1,9 bar). The distance between the tip of the air-polisher and the dental hard tissue was kept constant. A distance of 3–5 mm and an angle of 45° was used according to clinical procedures.

*Treatment with a rubber cup and polishing paste*. The sample surface was polished by a trained examiner (SF) for 5 seconds with a rubber cup (rubber cup blue Wkst205.3, Alfred Becht, Offenburg, Germany) and polishing paste (Plurapolish RDA 40, Pluradent, Offenbach, Germany) under moderate pressure (approximately 1.47 N) and circular movement. The examiner was trained to use always the same pressure by calibration using a letter scale. During perfomence of the study, a continuous recalibration was performed in order to ensure constant treatment conditions.

### Measurement methods

Two surface parameters were obtained (a) surface roughness and (b) substance loss. All measurement procedures were performed with contactless profilometry using a chromatic-confocal point topography sensor (vertical measuring range 300 μm, MikroProf 100, Acquire, FRT GmbH, Bergisch Gladbach, Germany) and measuring software for the analysis of the 3D scans (Mark III, FRT GmbH, Bergisch Gladbach, Germany). The measurement system was calibrated at the beginning of each measurement day according to the manufacturer’s instructions. Time points of measurement are given in Fig 1 (flow chart of study procedures).

#### Surface roughness

Surface roughness of all samples was measured thrice: (I) at baseline before treatment with the various cleaning methods (baseline); (II) after the treatment according to group assignment (Table 1; treatment) and (III) after cleaning in an ultrasonic bath for one minute (ultrasonography).

The standard measurement field size was set to 1 mm x 1 mm with a resolution in the x-direction of 500 points (2 μm distance/point) and in the y-direction of 200 lines (5 μm distance/line). The alignment of the individual samples in the coordinate system of the profilometer was performed via a limit stop on the measurement table. The coordinates of the markings previously set on the glass slides were recorded for each sample before the measurement was carried out. The distance between the start point of the measurement area and the reference point was recorded to measure the same area after each protocol step.

The surface roughness parameters sRa and sRz and the loss values were recorded, both measured in μm. Absolute values as well as differences between the measurements were both used for statistical analysis. Retained polishing paste could potentially fill cups and grooves of the surfaces and would than lead to incorrect positive effects of the polishing procedure with paste and rubber cup. Therefore, the differences between baseline/posttreatment and baseline/ultrasonic were compared. Only minor differences were found between both differences (for raw data see S1 and S2 Tables), indicating that polishing with a rubber cup does not lead to notable retention of polishing paste on the surface. Therefore, the data used for analysis are the difference between the time point after the final treatment in an ultrasonic bath and baseline.

To determine measurement consistency, one sample from each group was measured 10 times repeatedly under the same settings before the investigation. As a result, for flat samples, a deviation of 0.002 μm (sRa)/0.060 μm (sRz) for enamel and 0.001 μm (sRa)/0.281 μm (sRz) for dentin was found. For natural samples, a deviation of 0.101 μm (sRa)/0.946 μm (sRz) for enamel and 0.053 μm (sRa)/0.753 μm (sRz) for dentin was detected.

#### Substance loss

Substance loss was measured only on the flat samples as the precision and reliability of curved surfaces are lower than those of flat surfaces [7].

For measurment, the coverages on the reference surfaces were removed from the samples. All surfaces were checked (Leica Wild M3Z microscope, 6.5x to 16x magnification) for damage due to coverage removal and for coverage remnants.

On each sample, three parallel traces were made at intervals of 200 μm, 3 mm long, each. Half was placed on the reference area, while the other half was localized on the test area. On each trace, two regression lines were constructed, both on the reference area and the test area, both at least 0.3 mm off the border between the reference and test area and 0.5 mm in length. The vertical distance between the two regression lines was defined as the step height between the reference area and the test area. The mean of the three traces of one sample was defined as substance loss per sample (measured in μm) and was used for analysis.

### Statistics

For descriptive analysis, values are given as the mean ± standard deviation (SD). Boxplots were used for graphical presentation. To ensure a normal distribution, the data were transformed to a logarithmic scale of 10 for further analysis. Paired T-tests were used to compare the roughness values after different treatments within each group. Linear regression models were applied for comparisons between the groups for roughness parameters as well as the mean loss. In subsequent pairwise comparisons, the multiple testing was corrected according to the method of Scheffé. In addition, an interaction analysis was carried out for the roughness parameters and the mean loss values in the dentin to separate the individual effects of the curette, air-polishing, and rubber cup. All data were analyzed using the statistical software STATA (Version 17.0; College Station, TX, USA). The level of statistical significance was set to 0.05.

#### Sample size and power calculation

The sample size calculation was based on the current literature on roughness values after surface finishing assuming α = 0.05 and β = 0.2 with G*Power 3.1.9.4. In one study, root surfaces of bovine teeth (planar) were treated with different instruments for mechanical cleaning. After a 30-second application of a hand curette, there was an increase in roughness (Ra) compared to the baseline of 0.114±0.230 [8]. In another study, only the absolute roughness was measured (human samples, natural surfaces), but not the differences. However, this study compared different cleaning methods, similar to this study. It showed a difference (Ra) for dentin between the scaler (in cleaning comparable with the curette used in the present study) and air-polishing of 0.3, between scaler and polish of 0.6, and between polish and air-polishing of 1.0. In this work, no measure of scattering was given [9]. For enamel, smaller changes are expected due to the hardness of the tooth surface. In the study by Haas (2018) [9], differences between 0.1 and 0.3 were found when comparing the abovementioned treatment methods. In a dissertation from the University of Giessen [10], after treatment of prepared root surfaces (dentin, flat samples, human) with air-polishing (various powders, glycine, erythritol), changes in roughness (sRa) ranged from 0.013 to 0.381. The standard deviations were approximately 0.15. In enamel, a change between 0.026 and 0.207 was found. Here, the standard deviation was also 0.15. If a change due to the treatment (baseline to posttreatment) of 0.15 with a standard deviation of 0.15 is considered clinically relevant, then the number of cases per group is 10. If these values are considered differences between the groups, the number of cases is 17. Taking potential failures and sample losses into account, a sample size of 20 per group was calculated. With a group number of 8 per substrate and surface condition, 160 samples per experiment and thus a total of 320 enamel and 320 dentin samples were calculated.

## Results

### Roughness parameters

#### Enamel samples

For enamel samples, only minor changes in roughness values were found, both with flat and natural surfaces. On flat enamel samples, sRa increased significantly for most treatments except for rubber cup (3), curette/rubber cup (5), and the combination of three (7). sRz increased significantly for all groups on flat enamel samples except for air-polishing (2) and rubber cup (3). The highest value was found for the currette (1.69±1.72). For natural enamel samples, the curette led to a significant decrease in sRz, while a significant increase was found for air-polishing (2). The combination of all three (7) led to the highest decrease in sRz with a high standard deviation at the same time (-5.616±10.231). Details regarding enamel outcomes and data concerning different time points of measurement as well as significant values for all treatment groups can be found in the S1–S5 Tables.

#### Dentin samples

**Fig 3:**
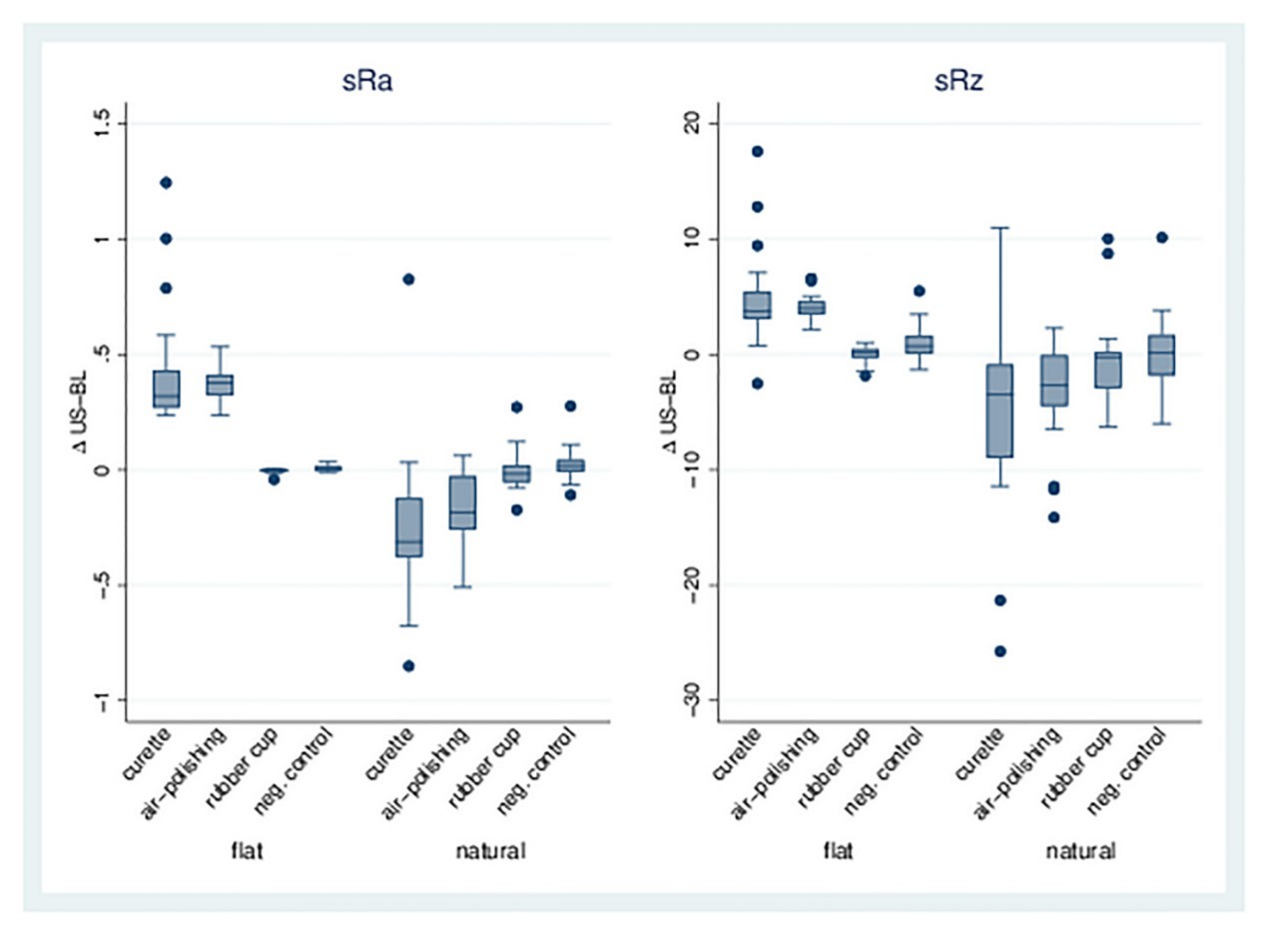
sRa and sRz for single treatments. Boxplots for dentin surfaces (flat/natural) with differences for sRa/sRz of treatment/baseline for treatment Groups 1, 2, 3, and 8 (negative control). In groups 3 and 8, only a smaller number of measurements could be performed because the adhesive bond between the sample and the slide had failed.

*Single treatments*. For the curette (1) and air-polishing (2) treatments, there was a significant increase in sRa on flat dentin surfaces compared to the control group (8). In contrast, a decrease in sRa for Groups 1 and 2 was observed on natural dentin surfaces. Likewise, sRz increased significantly on flat dentin surfaces after curette or air-polishing, whereas a significant reduction was observed on the natural dentin surfaces for the same treatments. The rubber cup (3) did not lead to a change in either sRa or sRz on flat or natural dentin surfaces. See also Table 2, Figs 3 and 5).

**Table 2:** sRa and sRz for single treatments and negative control.

Group	Treatment	sRa Flat Surface			sRa Natural Surface			sRz Flat Surface			sRz Natural Surface		
		N	Δ sRa Ultrasonic-Baseline		N	Δ sRa Ultrasonic-Baseline		N	Δ sRz Ultrasonic-Baseline		N	Δ sRz Ultrasonic-Baseline	
1	Curette	20	0.431*	±0.273	20	-0.256*	±0.327	20	4.897*	±4.285	20	-5.220*	±8.290
2	Air-polishing	20	0.371*	±0.066	20	-0.160*	±0.154	20	4.170*	±1.227	20	-3.431*	±4.508
3	Rubber-cup	20	-0.004	±0.011	19	-0.003	±0.090	19	-0.061	±0.780	19	-0.337	±3.933
8	Negative control	20	0.007*	±0.013	19	0.026	±0.080	20	0.992*	±1.580	19	0.320	±3.469

Mean values ± standard deviations for sRa and sRz in μm for the difference (Δ) ultrasonic baseline for flat and natural surfaces of dentin for treatment Groups 1, 2, 3, and 8,

*intragroup differences *p<0*.*05*.

**Fig 4:**
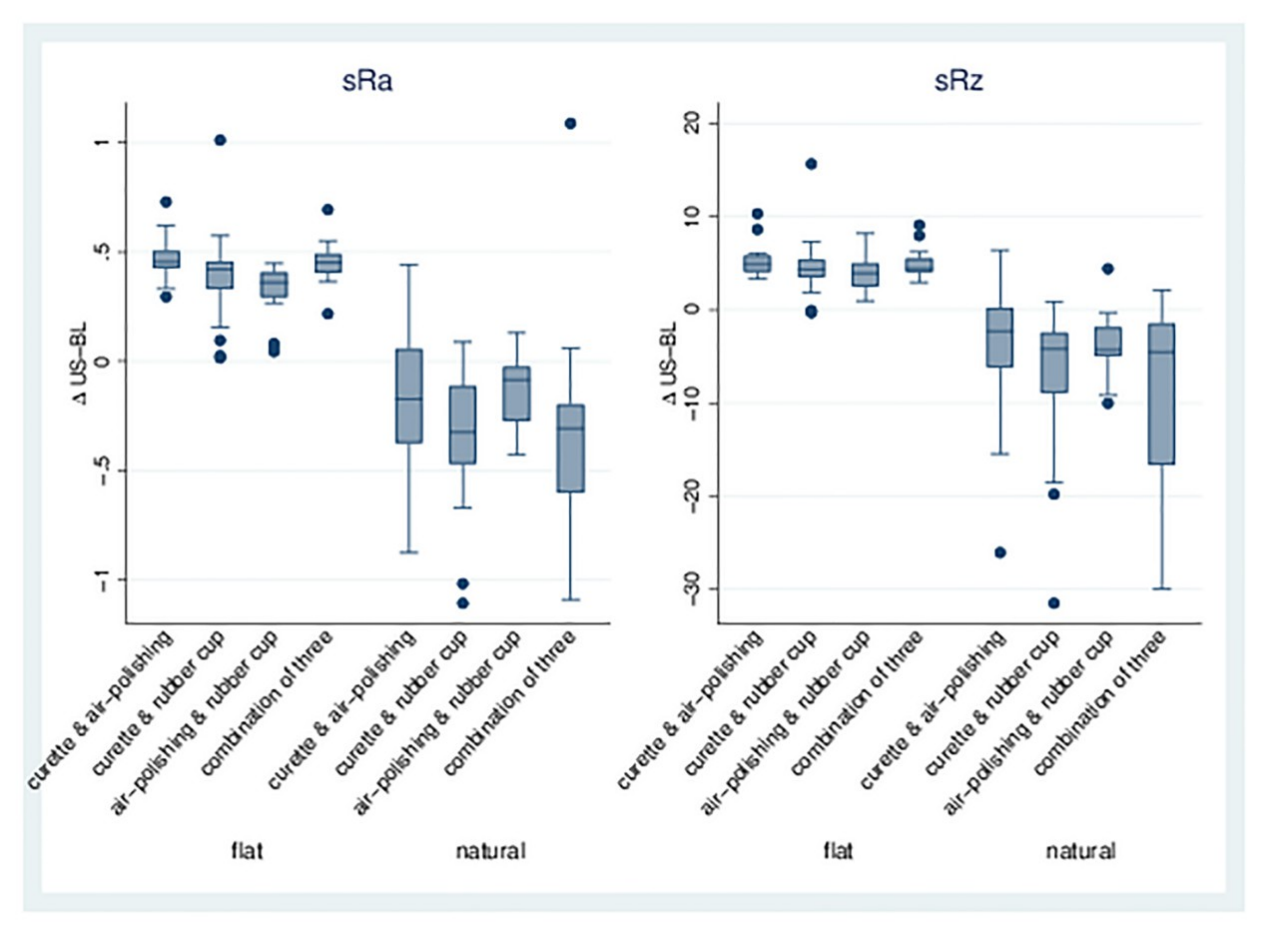
sRa and sRz for combinations of treatments. Boxplots for dentin surfaces (flat/natural) with differences for sRa/sRz of treatment-baseline for treatment Groups 4, 5, 6, and 7.

**Fig 5:**
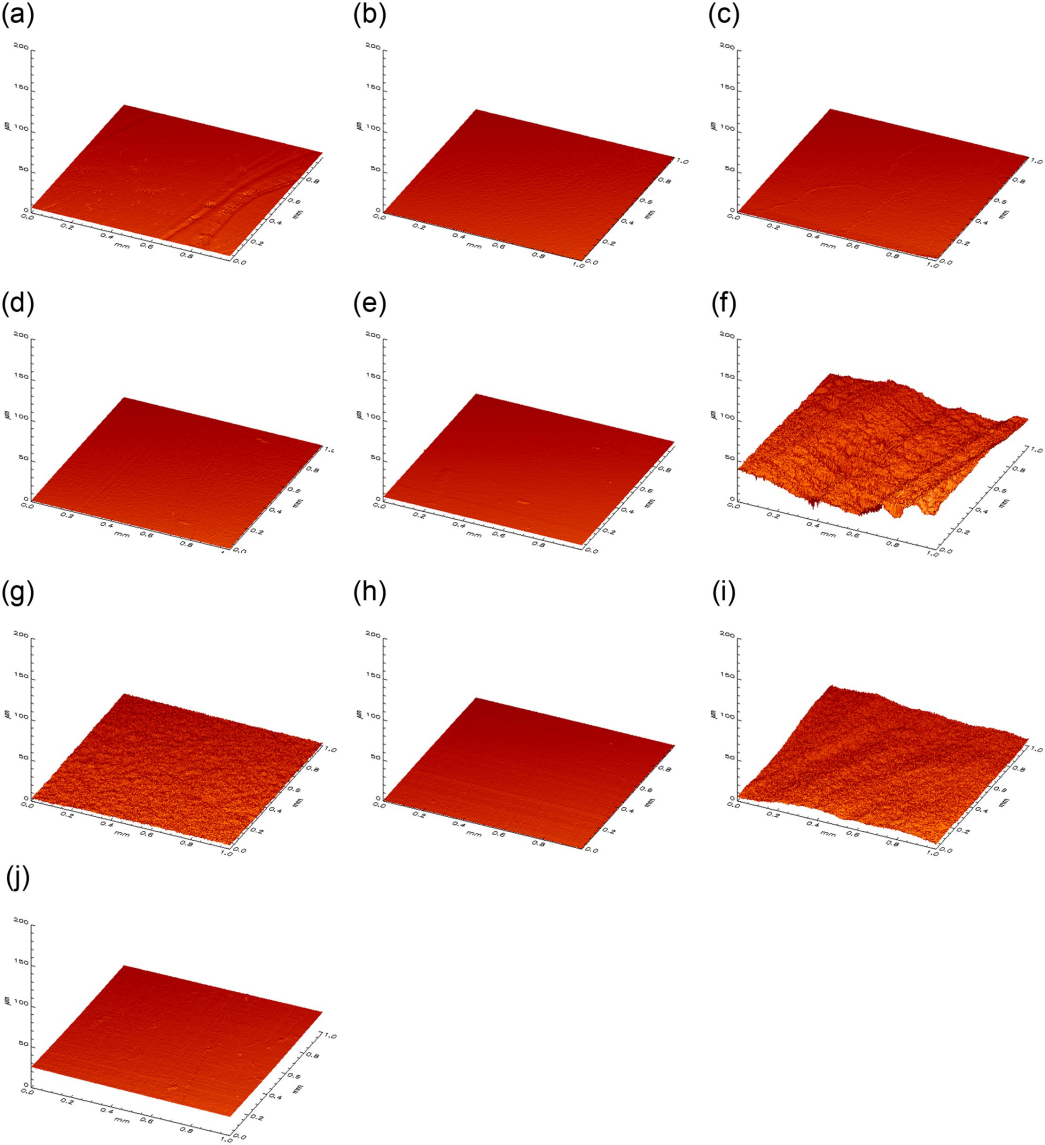
3D visualization of the surface roughness profile. Depiction of the measuring area (1 mm x 1 mm) of flat samples of enamel (a-e) and dentin (f-j) in μm. a/f = curette, b/g = air-polishing c/h = rubber cup, d/i = combination of three, e/j = negative.

*Combination of treatments*. The combination of different treatments (Groups 4–7) led to a significant increase in sRa and sRz on flat dentin samples, whereas a significant decrease was found for natural surfaces. Air-polishing/rubber cup (6) showed the least increase or decrease; all combinations with the curette (4, 5, 7) showed greater effects on roughness parameters (Table 3, Figs 4 and 5).

**Table 3:** Combinations of different treatments.

Group	Treatment	sRa Flat Surface			sRa Natural Surface			sRz Flat Surface			sRz Natural Surface		
		N	Δ sRa Ultrasonic-Baseline		N	Δ sRa Ultrasonic-Baseline		N	Δ sRz Ultrasonic-Baseline		N	Δ sRz Ultrasonic-Baseline	
4	Curette/ air-polishing	20	0.468*	±0.099	20	-0.193*	±0.333	20	5.132*	±1.730	20	-3.942*	±7.364
5	Curette/ rubber cup	19	0.390*	±0.223	20	-0.357*	±0.311	19	4.473*	±3.448	20	-7.086*	±7.910
6	Air-polishing/ rubber cup	20	0.322*	±0.121	18	-0.137*	±0.171	20	3.805*	±1.758	18	-3.688*	±3.299
7	Combination of three	20	0.451*	±0.092	19	-0.337*	±0.483	20	4.932*	±1.503	19	-7.664*	±8.763

Mean values ± standard deviations for sRa and sRz in μm for the difference (Δ) ultrasonic baseline for flat and natural surfaces of dentin for treatment Groups 4, 5, 6, and 7,

*intragroup differences *p<0*.*05*.

### Substance loss

**Fig 6:**
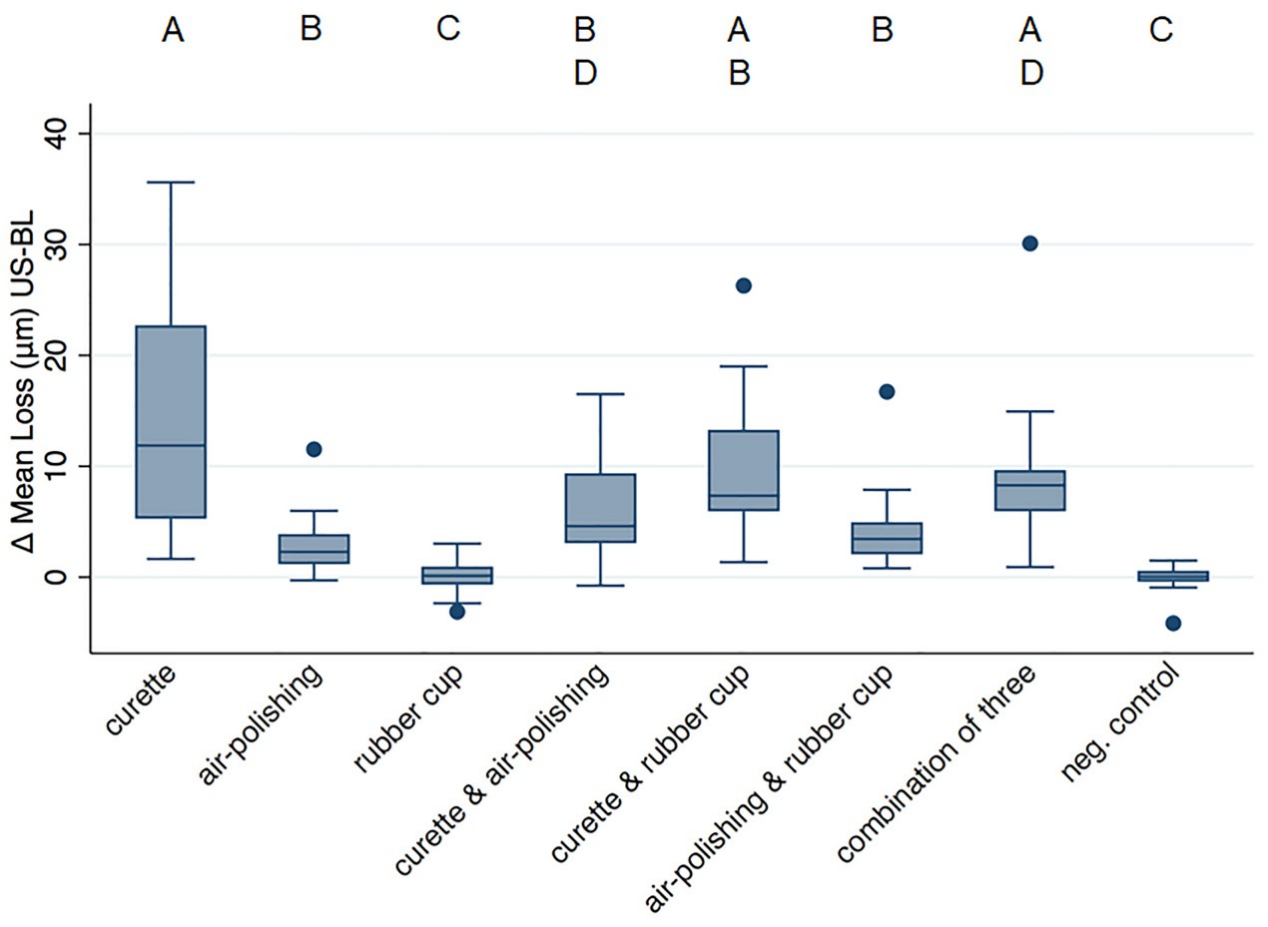
Surface loss values (μm) obtained from flat dentin samples. The different letters above indicate differences between groups.

Loss values are given as μm in the following text and depicted in Fig 6. Full data can be found in S3 Table. Surface loss measurement has only been performed on flat surfaces for data validity reasons. In enamel, none of the treatments provoked any tissue loss (*p>0*.*05* for all comparisons to the negative control). In dentin, significant losses (compared to negative control *p<0*.*0001*) were induced by treatments including the curette alone (14.9±10.5) or in any combination (minimal loss 6.2±4.9). Air-polishing alone (2.9±2.7) and in combination with rubber cup and paste (4.2±3.5) also created significant loss (compared to the negative control *p = 0*.*002*), but less than the curette; rubber cup polishing on its own showed no loss (*p>0*.*05*).

### Interaction analysis

To separate the individual effects of the curette, air-polishing, and rubber cup, an interaction analysis was carried out for the roughness parameters and the mean loss values in dentin. For the different ultrasonic-baseline, we observed comparable results in sRa-values for curette and air-polishing for flat samples (both 0.8 in logarithmic scale), while rubber cup showed lower values (0.5). The sRz values for flat samples as well as both sRz- and sRa-values for natural samples showed comparable results for all three techniques. For the flat samples, we observed higher mean loss values for the curette (9.9), while air-polishing (5.4) and rubber cup (5.3) were comparable. In natural samples, the effect of air-polishing (17.4) was slightly higher than that for rubber-cup (11.2) but lower than for the curette (22.3).

## Discussion

This *ex vivo* study investigated changes in surface roughness parameters (sRa, sRz) after different cleaning procedures on a total of 640 samples, including flat and natural enamel and dentin surfaces. The main findings were that the sRa values of flat enamel and dentin samples increased after all treatments except for rubber cup. For natural samples, more varying results were found due to particularities in the measurement method [11]; therefore, these results obtained from natural surfaces must be interpreted with care, however, more closely approximate the in vivo situation.

On dentin, sRa decreased after any treatment, while enamel showed an increase in roughness (sRa), except for rubber cup polishing. For sRz, the curette reduced this roughness parameter on enamel, while air-polishing led to an increase. Concerning substance loss in enamel, none of the tested methods led to measurable tissue loss. In dentin, air-polishing caused only 50% or less tissue loss compared to the curette. In principle, it seems consistent that due to the different properties of enamel and dentin, more changes were visible in the dentin during the present study. Enamel consists almost exclusively of inorganic materials, which are mainly carbonated hydroxyapatite crystals [12]. In contrast, dentin contains approximately 50 vol % carbonated hydroxyapatite minerals and 30 vol % collagen, which leads to a lower hardness of the substrate and thus less resistance to abrasion, and changes in the surface morphology occur more quickly [12]. This explains why greater changes were found for roughness parameters and substance loss in dentin than in enamel.

Based on the results, the null hypothesis that there are no differences in roughness after treatment with different cleaning methods was largely rejected but must be differentiated and considered in detail. Although minor changes in enamel were measurable, their small extent does not appear relevant, as the largest difference was found with the combination of curette, air-polishing, and rubber cup with only 0.054 μm (±0.056).

Looking at the comparability of the results in the literature, it is noticeable that there is only one comparable study that investigated the use of air-polishing with erythritol powder and investigated the surface roughness [13]. Here, the natural surfaces (enamel/dentin) of third molars were analyzed. However, in the respective *in vitro* study, erythritol powder with a reduced particle size of 7 μm was used, other than 14 μm in our study. The particle diameter has a significant influence on the abrasion properties of the powder [14], and the results may not be directly comparable. Nevertheless, in the mentioned study for enamel, no significant differences were found between different treatments for Rz. For dentin, a reduction in Rz was found after the use of the curette and rubber cup, while air-polishing did not show any differences. This in part supports our findings, as sRz decreased for all treatments on natural samples in our study. Another *in vitro* study by Sultan et al. investigated the surface roughness of root cementum after 20 s of air-polishing with glycine powder (45–60 μm particle size) and found a mean Ra of 0.5 μm for air-polishing only compared to 1.29 μm after the use of a curette [15]. Despite the use of a different powder substrate and the longer application time, the roughness for air-polishing is in a similar range to our results, while the curette produced a significantly higher roughness in the respective study. Here, the curette was used “until a smooth surface was obtained visually” [15]. The lack of standardization of this procedure could explain higher roughnesses, particularly due to more overlapping strokes.

When interpreting the results of the present study, it may be necessary to pay particular attention to the individual informative value of different measurement parameters. sRa shows the arithmetic mean of all peaks and lows of the profile in the selected area of 1 mm x 1 mm. Increasing or decreasing values are therefore less suitable for detecting particularly high peaks, as these could be mathematically neutralized by equally high lows. In contrast, the sRz value shows the quotient of amplitudes in five individual areas within the measuring area of 1 mm x 1 mm and is thus more capable of depicting pronounced deflections of the profile. For both the sRa and the sRz value differences in direction of roughness change were found between the natural and the flat surfaces. This is because the polised surfaces show very low roughness values, which can hardly be reduced by the treatement procedures. In contrast, the natural surfaces have an inherent roughness, which can be changed in both directions by the treatements. Basing on the mentioned detection potencies of sRa and sRz, for natural surfaces the sRz could be more relevant, as single peaks will be levelled off by the measurement principle. For flat surfaces, the sRa seems to give values that are more informative. Therefore, the direction of the changes cannot directly be compared between flat and natural surfaces. Nevertheless, the measured changes seem to provide indications for the different influence of the respective treatment on the surface. With natural surfaces, on the other hand, profilometry as an examination method has an increased risk of bias due to the natural curvature of the surface. The results are highly dependent on the surface that is approached to be optically measured, so in the case of irregular surfaces, even small displacements of the measuring point can lead to large differences here. Therefore, the occurrence of a measurement error is more likely with natural samples compared to flat surfaces, not least due to the curvature of the surface. Therefore, flat samples appear to be more meaningful for assessing the clinical relevance of the results.

For loss values, the present study was able to impressively show how invasive the use of the curette and its combination with other cleaning methods can be. Comparable results were found in other studies [5,16–18]. On the other hand, the application of air-polishing as well as rubber cup and paste on dentin resulted in only a slight loss of substance (noticeably less than 10 μm). Considering that professional tooth cleaning procedures are carried out several times a year, the regular use of curettes can quickly lead to a high loss of dental hard tissue, especially in dentin. For substance loss, more studies that are comparable are available in the literature. One recent study by Kröger et al. [16] compared the effects of glycine and erythritol powder on dentin and found no differences between the two types of powder. Furthermore, it was shown that air-polishing with low abrasive powders caused detectable but minor substance loss in dentin, confirming the findings of the present investigation. Compared to the use of sodium bicarbonate, the loss values are found to be significantly lower [5,17,19]. This effect is mainly explained by the properties of the other substrate used. Due to its higher hardness, crystalline structure, and larger particle size, sodium bicarbonate leads to significantly higher abrasion and is therefore only recommended for use on intact enamel but not on dentin [20]. Another study by Sahrmann et al. showed a significantly lower defect depth and defect volume when glycine powder was used for 5 sec compared with bicarbonate powder [17]. However, since data were given here in mm and mm^3^, very small numbers in the μm range are difficult to compare. Due to the broad spectrum of different experimental setups of the existing *in vitro* studies and the use of different powder substrates, it seems necessary to conduct additional future studies, in particular with repeated use of air-polishing in order to get an idea of cumulative effects.

Within the present study, no major effect on substance loss was observed for the cup and paste polishing step. This is confirmed by the literature [13,21]. Camboni and Donnet were able to show with scanning electron microscopy images that at least on enamel microcavities are more clearly exposed by air-polishing with erythritol and that a kind of flattening of these areas occurs due to polishing [3]. The working group suspected that the polishing paste abraded tooth debris, which might smear existing cavities and irregularities and thus supposedly mask the roughness. This hypothesis could be clearly negated by our investigation, as no relevant increase in roughness for dentin or enamel could be detected during subsequent cleaning with an ultrasonic bath. It should be emphasized that this study is the first to investigate this question.

## Conclusion

The effects of different professional cleaning procedures on surface roughness and substance loss were found to be greater for dentin than for enamel. While roughness for flat enamel and dentin surfaces as well as natural enamel tends to increase, natural dentin surfaces showed reduced surface roughness. Their extent for enamel was measurable but of limited clinical relevance. A subsequent polishing step with a rubber cup and paste did not affect the surface roughness and therefore seems unnecessary as a final procedure.

## Supporting information

S1 Table sRa data for all treatment groups on enamel and dentin. Mean differences and standard deviation for sRa of different time points (treatment-baseline, ultrasonic-baseline, ultrasonic-treatment) for flat and natural surfaces of dentin and enamel of each treatment group.
(DOCX)

S2 Table sRz data for all treatment groups on enamel and dentin. Mean differences and standard deviation for sRz of different time points (treatment-baseline, ultrasonic-baseline, ultrasonic-treatment) for flat and natural surfaces of dentin and enamel of each treatment group.
(DOCX)

S3 Table Loss values in dentin and enamel for flat samples of each treatment group. Mean differences and standard deviation for loss between different time points (ultrasonic vs. baseline). Bold data indicate significance compared to the negative control.
(DOCX)

S4 Table Significance values for sRa data for all treatment groups on enamel (upper triangle) and dentin (lower triangle) for flat surfaces. Bold data indicate significance.
(DOCX)

S5 Table Significance values for sRz data for all treatment groups on enamel (upper triangle) and dentin (lower triangle) for flat surfaces. Bold data indicate significance.
(DOCX)
