# Supplementary material for: Impact of air-polishing using erythritol on surface roughness and substance loss in dental hard tissue: An ex vivo study
Source: PLoS One. 2024 Feb 26;19(2):e0286672. doi: 10.1371/journal.pone.0286672 (PMC10896509; doi:10.1371/journal.pone.0286672)
Supplement: S1 Table — Mean differences and standard deviation for sRa of different time points (treatment-baseline, ultrasonic-baseline, ultrasonic-treatment) for flat and natural surfaces of dentin and enamel of each treatment group. (DOCX) [file pone.0286672.s001.docx]

**S1 Table.** sRa data for all treatment groups on enamel and dentin.

| Group | Treatment | Substrate | Flat Surface | |  |  |  |  |  | Natural Surface | |  |  |  |  |  |
| --- | --- | --- | --- | --- | --- | --- | --- | --- | --- | --- | --- | --- | --- | --- | --- | --- |
|  |  |  | N | Δ sRa  Treatment-Baseline | | Δ sRa  Ultrasonic-Baseline | | Δ sRa  Ultrasonic-Treatment | | N | Δ sRa  Treatment-Baseline | | Δ sRa  Ultrasonic-Baseline | | Δ sRa  Ultrasonic-Treatment | |
| 1 | Curette | Enamel | 19 | 0.416 | ±0.046 | 0.035 | ±0.037 | -0.007 | ±0.022 | 20 | -0.054 | ±0.105 | -0.044 | ±0.104 | 0.011 | ±0.037 |
| 2 | Air-Polishing |  | 20 | 0.027 | ±0.022 | 0.033 | ±0.027 | 0.006 | ±0.014 | 20 | 0.081 | ±0.457 | 0.074 | ±0.041 | -0.007 | ±0.012 |
| 3 | Rubber-cup |  | 20 | 0.004 | ±0.162 | 0.004 | ±0.014 | 0.000 | ±0.009 | 20 | 0.008 | ±0.057 | -0.013 | ±0.079 | -0.021 | ±0.059 |
| 4 | Curette / air-polishing |  | 20 | 0.038 | ±0.027 | 0.037 | ±0.027 | -0.001 | ±0.004 | 18 | 0.133 | ±0.195 | 0.134 | ±0.206 | 0.001 | ±0.150 |
| 5 | Curette / rubber-cup |  | 19 | 0.031 | ±0.025 | 0.027 | ±0.028 | -0.004 | ±0.012 | 20 | 0.057 | ±0.160 | 0.069 | ±0.164 | 0.011 | ±0.050 |
| 6 | Air-polishing / rubber-cup |  | 20 | 0.033 | ±0.022 | 0.032 | ±0.022 | -0.001 | ±0.004 | 19 | 0.056 | ±0.038 | 0.043 | ±0.024 | -0.013 | ±0.025 |
| 7 | Combination of three |  | 19 | 0.057 | ±0.056 | 0.054 | ±0.056 | -0.003 | ±0.005 | 20 | 0.032 | ±0.186 | 0.035 | ±0.187 | 0.003 | ±0.018 |
| 8 | Negative control |  | 20 | 0.003 | ±0.007 | 0.009 | ±0.019 | 0.006 | ±0.019 | 20 | -0.002 | ±0.034 | -0.001 | ±0.035 | 0.002 | ±0.018 |
| 1 | Curette | Dentin | 20 | 0.400 | ±0.187 | 0.431 | ±0.273 | 0.031 | ±0.098 | 20 | -0.275 | ±0.319 | -0.256 | ±0.327 | 0.020 | ±0.051 |
| 2 | Air-Polishing |  | 20 | 0.388 | ±0.066 | 0.371 | ±0.066 | -0.018 | ±0.011 | 20 | -0.145 | ±0.153 | -0.160 | ±0.154 | -0.014 | ±0.049 |
| 3 | Rubber-cup |  | 20 | -0.004 | ±0.014 | -0.004 | ±0.011 | 0.000 | ±0.008 | 19 | -0.075 | ±0.065 | -0.003 | ±0.090 | 0.072 | ±0.099 |
| 4 | Curette /air-polishing |  | 20 | 0.475 | ±0.100 | 0.468 | ±0.099 | -0.007 | ±0.007 | 20 | -0.191 | ±0.336 | -0.193 | ±0.333 | -0.002 | ±0.010 |
| 5 | Curette / rubber-cup |  | 19 | 0.371 | ±0.191 | 0.390 | ±0.223 | 0.020 | ±0.052 | 20 | -0.367 | ±0.345 | -0.357 | ±0.311 | 0.011 | ±0.097 |
| 6 | Rubber-cup / air-polishing |  | 20 | 0.334 | ±0.125 | 0.322 | ±0.121 | -0.011 | ±0.013 | 18 | -0.161 | ±0.168 | -0.137 | ±0.171 | 0.025 | ±0.048 |
| 7 | Combination of three |  | 20 | 0.468 | ±0.090 | 0.451 | ±0.092 | -0.017 | ±0.018 | 19 | -0.322 | ±0.486 | -0.337 | ±0.483 | -0.014 | ±0.034 |
| 8 | Negative control |  | 20 | 0.009 | ±0.010 | 0.007 | ±0.013 | -0.002 | ±0.009 | 19 | 0.040 | ±0.143 | 0.026 | ±0.080 | -0.014 | ±0.084 |

Mean differences and standard deviation for sRa of different time points (treatment-baseline, ultrasonic-baseline, ultrasonic-treatment) for flat and natural surfaces of dentin and enamel of each treatment group.
