# Supplementary material for: Impact of air-polishing using erythritol on surface roughness and substance loss in dental hard tissue: An ex vivo study
Source: PLoS One. 2024 Feb 26;19(2):e0286672. doi: 10.1371/journal.pone.0286672 (PMC10896509; doi:10.1371/journal.pone.0286672)
Supplement: S2 Table — Mean differences and standard deviation for sRz of different time points (treatment-baseline, ultrasonic-baseline, ultrasonic-treatment) for flat and natural surfaces of dentin and enamel of each treatment group. (DOCX) [file pone.0286672.s002.docx]

**S2 Table.** sRz data for all treatment groups on enamel and dentin.

| Group | Treatment | Substrate | Flat Surface | |  |  |  |  |  | Natural Surface | | | | | |  |
| --- | --- | --- | --- | --- | --- | --- | --- | --- | --- | --- | --- | --- | --- | --- | --- | --- |
|  |  |  | N | Δ sRz  Treatment-Baseline | | Δ sRz  Ultrasonic-Baseline | | Δ sRz  Ultrasonic-Treatment | | N | Δ sRz  Treatment-Baseline | | Δ sRz  Ultrasonic-Baseline | | Δ sRz  Ultrasonic-Treatment | |
| 1 | Curette | Enamel | 19 | 1.690 | ±1.720 | 2.285 | ±3.874 | 0.595 | ±3.879 | 20 | -2.050 | ±2.801 | -1.597 | ±2.918 | 0.452 | ±1.175 |
| 2 | Air-Polishing |  | 20 | 0.319 | ±1.115 | 0.787 | ±2.010 | 0.468 | ±2.021 | 20 | 3.233 | ±5.719 | 2.831 | ±4.752 | -0.402 | ±2.287 |
| 3 | Rubber-cup |  | 20 | -0.251 | ±0.689 | -0.315 | ±0.570 | -0.064 | ±0.421 | 20 | -1.012 | ±2.131 | -0.764 | ±2.418 | 0.248 | ±1.208 |
| 4 | Curette / air-polishing |  | 20 | 0.038 | ±0.683 | 0.472 | ±0.517 | -0.211 | ±0.425 | 18 | 0.869 | ±3.180 | 0.721 | ±3.014 | -0.148 | ±0.711 |
| 5 | Curette / rubber-cup |  | 19 | 1.197 | ±1.357 | 0.890 | ±1.089 | -0.308 | ±0.928 | 20 | 0.615 | ±3.160 | 0.435 | ±2.756 | -0.180 | ±1.359 |
| 6 | Rubber-cup / air-polishing |  | 20 | 0.433 | ±0.427 | 0.545 | ±0.773 | 0.112 | ±0.775 | 19 | 2.347 | ±5.576 | 0.300 | ±2.516 | -2.048 | ±4.450 |
| 7 | Combination of three |  | 19 | 1.680 | ±1.756 | 1.117 | ±1.805 | -0.562 | ±1.084 | 20 | 0.325 | ±3.751 | 0.504 | ±3.928 | 0.179 | ±1.124 |
| 8 | Negative control |  | 20 | 0.621 | ±0.647 | 0.870 | ±0.821 | 0.250 | ±0.971 | 20 | -0.045 | ±1.333 | 0.281 | ±1.353 | 0.326 | ±0.729 |
| 1 | Curette | Dentin | 20 | 4.688 | ±2.607 | 4.897 | ±4.285 | 0.209 | ±2.090 | 20 | -3.527 | ±9.568 | -5.220 | ±8.290 | -1.693 | ±6.351 |
| 2 | Air-Polishing |  | 20 | 4.268 | ±1.055 | 4.170 | ±1.227 | -0.098 | ±0.406 | 20 | -1.213 | ±6.513 | -3.431 | ±4.508 | -2.219 | ±5.410 |
| 3 | Rubber-cup |  | 20 | 0.057 | ±1.382 | -0.061 | ±0.780 | -0.118 | ±1.169 | 19 | -3.436 | ±4.168 | -0.337 | ±3.933 | 0.026 | ±0.030 |
| 4 | Curette / air-polishing |  | 20 | 5.189 | ±1.747 | 5.132 | ±1.730 | -0.057 | ±0.431 | 20 | -3.858 | ±7.073 | -3.942 | ±7.364 | -0.084 | ±2.728 |
| 5 | Curette / rubber-cup |  | 19 | 4.551 | ±2.528 | 4.473 | ±3.448 | -0.788 | ±2.174 | 20 | -5.142 | ±7.390 | -7.086 | ±7.910 | -1.944 | ±5.549 |
| 6 | Rubber-cup / air-polishing |  | 20 | 3.917 | ±1.720 | 3.805 | ±1.758 | -0.112 | ±0.278 | 18 | -3.693 | ±4.270 | -3.688 | ±3.299 | 0.005 | ±3.489 |
| 7 | Combination of three |  | 20 | 5.173 | ±1.196 | 4.932 | ±1.503 | -0.241 | ±1.285 | 19 | -5.616 | ±10.231 | -7.664 | ±8.763 | -2.048 | ±3.475 |
| 8 | Negative control |  | 20 | 0.257 | ±0.713 | 0.992 | ±1.580 | 0.735 | ±1.495 | 19 | 0.447 | ±4.284 | 0.320 | ±3.469 | -0.128 | ±4.087 |

Mean differences and standard deviation for sRz of different time points (treatment-baseline, ultrasonic-baseline, ultrasonic-treatment) for flat and natural surfaces of dentin and enamel of each treatment group.
