# Supplementary material for: Impact of air-polishing using erythritol on surface roughness and substance loss in dental hard tissue: An ex vivo study
Source: PLoS One. 2024 Feb 26;19(2):e0286672. doi: 10.1371/journal.pone.0286672 (PMC10896509; doi:10.1371/journal.pone.0286672)
Supplement: S3 Table — Mean differences and standard deviation for loss between different time points (ultrasonic vs. baseline). Bold data indicate significance compared to the negative control. (DOCX) [file pone.0286672.s003.docx]

**S3 Table.** Loss values in dentin and enamel for flat samples of each treatment group.

| Group | Treatment | Substrate | Flat Surface | | | Natural Surface | | |
| --- | --- | --- | --- | --- | --- | --- | --- | --- |
|  |  |  | N | Loss Ultrasonic-Baseline | | N | Loss Ultrasonic-Baseline | |
| 1 | Curette | Enamel | 18 | 0.102 | ±0.305 | 20 | -1.466 | ±8.028 |
| 2 | Air-Polishing |  | 19 | 0.111 | ±0.318 | 19 | 0.990 | ±5.812 |
| 3 | Rubber-cup |  | 20 | 0.109 | ±0.348 | 15 | -0.565 | ±3.772 |
| 4 | Curette / air-polishing |  | 20 | 0.022 | ±0.496 | 15 | 4.063 | ±7.858 |
| 5 | Curette / rubber-cup |  | 19 | 0.088 | ±0.282 | 14 | -0.426 | ±8.378 |
| 6 | Rubber-cup / air-polishing |  | 18 | 0.128 | ±0.327 | 15 | -0.560 | ±7.394 |
| 7 | Combination of three |  | 19 | -0.023 | ±0.390 | 19 | -0.308 | ±11.723 |
| 8 | Negative control |  | 18 | 0.170 | ±0.481 | 14 | 1.337 | ±8.689 |
| 1 | Curette | Dentin | 20 | **14.911** | ±10.464 | 16 | **24.372** | ±21.223 |
| 2 | Air-Polishing |  | 20 | **2.875** | ±2.666 | 15 | 14.539 | ±27.165 |
| 3 | Rubber-cup |  | 20 | 0.052 | ±1.498 | 18 | 0.964 | ±11.408 |
| 4 | Curette / air-polishing |  | 20 | **6.219** | ±4.938 | 16 | 28.480 | ±39.301 |
| 5 | Curette / rubber-cup |  | 18 | **9.391** | ±6.251 | 17 | 17.286 | ±20.853 |
| 6 | Rubber-cup / air-polishing |  | 20 | **4.164** | ±3.529 | 14 | 7.736 | ±23.752 |
| 7 | Combination of three |  | 20 | **8.640** | ±6.149 | 17 | 18.824 | ±22.544 |
| 8 | Negative control |  | 20 | -0.027 | ±1.200 | 18 | 2.144 | ±6.979 |

Mean differences and standard deviation for loss between different time points (ultrasonic vs. baseline). Bold data indicate significance compared to the negative control.
