# Supplementary material for: Impact of air-polishing using erythritol on surface roughness and substance loss in dental hard tissue: An ex vivo study
Source: PLoS One. 2024 Feb 26;19(2):e0286672. doi: 10.1371/journal.pone.0286672 (PMC10896509; doi:10.1371/journal.pone.0286672)
Supplement: S4 Table — Bold data indicate significance. (DOCX) [file pone.0286672.s004.docx]

**S4 Table**. Significance values for sRa data for all treatment groups on enamel (upper triangle) and dentin (lower triangle) for flat surfaces.

| **sRa** | |  |  |  |  |  |  |  |  |  |
| --- | --- | --- | --- | --- | --- | --- | --- | --- | --- | --- |
|  | **Enamel** | **Flat surface** |  | **Treatment - Baseline** |  |  |  |  |  |  |
| **Dentin** |  |  |  |  |  |  |  |  |  |  |
| **Treatment** | |  | **Curette** | **Air-polishing** | **Rubber cup** | **Curette / Air-polishing** | **Curette / Rubber cup** | **Air-polishing / Rubber cup** | **Combination of three** | **Negative control** |
|  | | **Group** | **1** | **2** | **3** | **4** | **5** | **6** | **7** | **8** |
| **Curette** | | **1** |  | 0,964 | **0,002** | 1,000 | 1,000 | 1,000 | 0,868 | **0,000** |
| **Air-polishing** | | **2** | 1,000 |  | **0,009** | 0,823 | 0,995 | 0,993 | 0,142 | **0,000** |
| **Rubber cup** | | **3** | **0,000** | **0,000** |  | **0,000** | **0,001** | **0,001** | **0,000** | 1,000 |
| **Curette / Air-polishing** | | **4** | 0,654 | 0,208 | **0,000** |  | 0,995 | 0,996 | 0,963 | **0,000** |
| **Curette / Rubber cup** | | **5** | 0,998 | 0,989 | **0,000** | 0,640 |  | 1,000 | 0,590 | **0,000** |
| **Air-polishing /**  **Rubber cup** | | **6** | 0,975 | 0,863 | **0,000** | 0,145 | 1,000 |  | 0,602 | **0,000** |
| **Combination of three** | | **7** | 0,707 | 0,245 | **0,000** | 1,000 | 0,673 | 0,166 |  | **0,000** |
| **Negative control** | | **8** | **0,000** | **0,000** | 0,101 | **0,000** | **0,000** | **0,000** | **0,000** |  |

Bold data indicate significance.
