# Supplementary material for: Impact of air-polishing using erythritol on surface roughness and substance loss in dental hard tissue: An ex vivo study
Source: PLoS One. 2024 Feb 26;19(2):e0286672. doi: 10.1371/journal.pone.0286672 (PMC10896509; doi:10.1371/journal.pone.0286672)
Supplement: S5 Table — Bold data indicate significance. (DOCX) [file pone.0286672.s005.docx]

**S5 Table**. Significance values for sRz data for all treatment groups on enamel (upper triangle) and dentin (lower triangle) for flat surfaces.

| **sRz** | |  |  |  |  |  |  |  |  |  |
| --- | --- | --- | --- | --- | --- | --- | --- | --- | --- | --- |
|  | **Enamel** | **Flat surface** |  | **Treatment - Baseline** |  |  |  |  |  |  |
| **Dentin** |  |  |  |  |  |  |  |  |  |  |
| **Treatment** | |  | **Curette** | **Air-polishing** | **Rubber cup** | **Curette / Air-polishing** | **Curette / Rubber cup** | **Air-polishing / Rubber cup** | **Combination of three** | **Negative control** |
|  | | **Group** | **1** | **2** | **3** | **4** | **5** | **6** | **7** | **8** |
| **Curette** | | **1** |  | **0,040** | **0,000** | 0,226 | 0,971 | **0,025** | 1,000 | 0,176 |
| **Air-polishing** | | **2** | 0,968 |  | 0,913 | 0,853 | 0,606 | 0,999 | 0,186 | 0,966 |
| **Rubber cup** | | **3** | **0,000** | **0,000** |  | **0,005** | **0,016** | 0,175 | **0,001** | 0,060 |
| **Curette / Air-polishing** | | **4** | 0,999 | 0,439 | **0,000** |  | 0,988 | 0,949 | 0,674 | 1,000 |
| **Curette / Rubber cup** | | **5** | 1,000 | 1,000 | **0,000** | 0,993 |  | 0,744 | 0,998 | 0,962 |
| **Air-polishing /**  **Rubber cup** | | **6** | 0,822 | 0,995 | **0,000** | 0,437 | 0,980 |  | 0,236 | 0,996 |
| **Combination of three** | | **7** | 1,000 | 0,322 | **0,000** | 1,000 | 0,994 | 0,404 |  | 0,577 |
| **Negative control** | | **8** | **0,000** | **0,000** | 0,659 | **0,000** | **0,000** | **0,000** | **0,000** |  |

Bold data indicate significance.
